# Preparation of a Fast Water-Based UV Cured Polyurethane-Acrylate Wood Coating and the Effect of Coating Amount on the Surface Properties of Oak (*Quercus alba* L.)

**DOI:** 10.3390/polym11091414

**Published:** 2019-08-28

**Authors:** Jin Wang, Huagui Wu, Ru Liu, Ling Long, Jianfeng Xu, Minggui Chen, Hongyun Qiu

**Affiliations:** 1 Research Institute of Forestry New Technology, Chinese Academy of Forestry, Haidian, Beijing 100091, China; 2 Kingdecor (Zhejiang) Co., Ltd., Quzhou 324000, China; 3 Research Institute of Wood Industry, Chinese Academy of Forestry, Haidian, Beijing 100091, China; 4 Jiangsu Himonia Technology Co., Ltd., Jurong 212400, China

**Keywords:** water-based UV curing coating, coating amount, surface properties, polyurethane-acrylate, oak (*Quercus alba* L.)

## Abstract

A fast water-based ultraviolet light (UV) curing polyurethane-acrylate (PUA) wood coating was prepared in the laboratory, and applied on oak (*Quercus alba* L.) at different coating amounts. The PUA wood coating can be fast cured within 22 min, which highly improved the drying speed compared to normal water-based wood coatings (often higher than 35 min). The coating amounts affected the coating properties after curing on oak. With the increase of coating amount, the adhesion, hardness and gloss value of surface increased to different extents. Meanwhile, the surface of sample became smooth gradually because the voids of the oak were filled. Thus, higher coating amount resulted in better coating properties. However, no significant increase of penetration depth was found. During curing, the hydroxyl groups of the wood reacted with the coating. The optimal parameter in this study was the coating amount of 120 g/m^2^, where the adhesion reached 1 (with 0–5% cross-cut area of flaking along the edges), with the hardness of 2H and the gloss of 92.56°, which met the requirement of Chinese standard GB/T 18103–2013, and could be used for engineered wood flooring.

## 1. Introduction

In recent years, with the improvement of environmental protection regulations and people’s awareness, water-based wood coatings have become a research hotspot [1,2,3,4,5]. Water-based polyurethane acrylate (PUA) coatings are widely used owing to their wear-resistance, stain resistance, and ease of use [6]. They can form a variety of hydrogen bonds between molecular chains due to their functional acrylic groups and urethane bonds, which provides the coatings with weather resistance, solvent resistance, comprehensive mechanical properties and excellent appearance on wood products [7,8,9,10]. At the same time, the molecular structure and functionality can be adjusted to satisfy the different needs of furniture, cabinets, office and laboratory equipment. In addition, during film formation, little or no volatile organic compounds (VOCs) evaporate into the atmosphere, therefore they can be considered low cost and pollution-free. 

However, a problem that has restricted the application of water-based coatings is their relatively slow drying rate compared with solvent-based coatings. It is known that the latent heat of water is much higher than that of organic solvents, such as benzene, xylene, etc. Besides, some properties of water-based coatings are not comparable to those of solvent-based coatings, like low hardness, and gloss. To expand the application of coatings to other areas, ultraviolet light (UV) curing technology is used in the manufacture of water-based coatings for fast curing [11,12,13]. UV cured coatings use high energy UV lights as sources, which can form coating films via self-polymerization [14]. Most UV-curable resins consist of multifunctional acrylate monomers and oligomers associated to an aromatic ketone that generates upon UV-exposure free radicals which will initiate the crosslinking polymerization [15]. Therefore, the PUA could meet these requirements. Some kinds of water-based UV curing PUA coatings have been investigated [15,16]. However, up to present, the water-based UV curing coatings have not really been applied on wooden products due to the limitations of the coating film properties. 

It is well known that the properties of coatings are affected by many factors, such as the environment, degree of wetting between the coating and the substrate, chemical or physical effects, surface properties of the substrate, and so on [17,18,19,20,21,22]. Yang et al. [23] found that different environments could cause differences in the chemical composition on the surface of the PUA painting film and the surface morphology was thus different. Masson et al. [16] also found that basic coating performance features such as the drying speed were highly dependent on the temperature and film thickness.

Accordingly, many researchers have investigated the basic properties of the resin particles and the impact of paint in different environments [24,25,26,27,28,29]. However, the influence of the coating amount on the resulting surface performance of the coating has been less investigated, let alone for fast water-based UV curing PUA wood coatings. Coating amount, as a very important factor, affects the performance of cured coatings very much. At low coating amount, the coating thickness is thin and insufficient coverage will be obtained, while with an excessive amount of coating, drying defects such as wrinkles, and bubbling may occur. Therefore, in this paper, a kind of fast water-based UV curing coating was synthesized in the laboratory, and applied on oak for its wide application in furniture, flooring and other industries due to its resource advantages and good decorative properties in the solid wood market [30,31,32]. The effect of coating amount on the surface properties such as the surface morphology, chemical changes, permeability and surface energy was studied, with the aim to provide some basic data and reference for the application of the fast water-based UV curing wood coatings.

## 2. Materials and Methods 

### 2.1. Materials

Oak (*Quercus alba* L.) wood which originated from Fujian Province in the southeast of China was purchased from a Beijing home building materials market. The wood was dried to a 8–10% moisture content prior to use. The oak was then sawn into slabs with dimensions of 630 mm (L) × 120 mm (T) × 20 mm (R). The slabs were sanded with 240 mesh sandpaper and the dust was removed to ensure that the substrate surface was clean. The reagents for the fast water-based UV curing PUA wood coating are listed in Table 1. All abbreviations concerning the reagents used are presented in parentheses in Table 1.

### 2.2. Synthesis of the Fast Water-Based UV Curing PUA Coating

The fast water-based UV curing PUA coating was prepared based on our previous study [33]. According to Table 1, the first step was to prepare a pre-polyacrylate emulsion. Monomers (24 g of AA, 4 g of ST, and 7 g of EA) were blended with 169 g of distilled water in a three-neck glass flask at 150 r/min. Afterwards, the initiator (8 g of APS) was added at 10 wt%. For the next 70–90 min, the rest of the monomers (96 g of AA, 16 g of ST, and 133 g of EA) was added dropwise into the reactor. Afterwards, the rest of the initiator (32 g of APS) and 36 g of KH550 were added dropwise into the reactor within 90–110 min. The reactor was heated up to 85 °C and held at this temperature for 4 h. The next step was to graft the pre-polyurethane onto the pre-polyacrylate chain. TDI (100 g), PEG-400 (200 g) and DBTD (8 g) were dissolved in *N*-methyl-2-pyrrolidone solvent and blended into a three-neck glass flask at 150 r/min. The reactor was heated to 40 °C. While stirring, HEA (66 g) was dripped into the reactor within 2 h. The pre-polyurethane was thus obtained. Afterwards, both the pre-polyacrylate and pre-polyurethane were mixed in the three-neck glass flask and blended at 150 r/min. The reaction mixture was heated up to 85 °C and kept at that temperature for 2 h. The pre-PUA emulsion was obtained after cooling and filtration. Defoamer agent (0.2%), photoinitiator (2%), and HDDA diluting agent (1.5%) were added into the emulsion at 100 r/min for 10 min. Then, the water-based UV curing PUA coating was obtained. After good dispersion, the coating was prepared and stored in a black bottle against light.

### 2.3. Coating on a Wood Sample

The coating amount was determined as a variable, and an uncoated wood slab was used as a control. The wet coating coating amount weights were 12, 60, and 120 g/m^2^, respectively. The coating process was carried out by on an auto-coating rolling machine bracket with pressure. The coating speed was 80 mm/s. The drying temperature of the hot air dryer used in this test was determined to be 35 °C. The dryer mesh was subjected to a drying cycle of 2 min with 10 drying cycles, where the removal of water was achieved. Then, the coating it was cured by a UV curing machine. The photocuring energy was 250–350 mJ/cm^2^ for 2 min.

### 2.4. Characterizations and Tests

The solid content of PUA was measured according to Chinese Standard GB/T 1725–2007 by drying the PUA coating at 125 °C for 1 h based on the residual percent of the coating. The viscosity was determined by a viscometer (NDJ-5S, Shanghai Pingxuan Scientific Instrument Co., Ltd., Shanghai, China). The average particle size of PUA was measured by a laser scattering particle distribution analyzer (MPT-Z, Malvern, London, UK). 

The macro-structure was observed by an optical stereomicroscope (Leica S9 series, Jena, Germany). The microstructures of the above samples were observed by scanning electron microscope (SEM, JEOL JSM-6301F, Tokyo, Japan) with an acceleration voltage of 20 kV. The samples were sputter-coated with gold.

The dried coating thickness was measured by an ultrasonic velocity gauge (AT-140S, Guangzhou Amittari Design, Co. Ltd., Guangzhou, China). The throughout-dry state of the coating after 22 min of curing was tested following the ISO 9117:1990 standard with a plunger machine. The adhesion classification of coating films with wood was measured according to the ISO 2409:2013 standard method by a cross-cut test. The gloss values of the coatings were determined by the ISO 2813:2014 method using a geometry of 60°. Coating hardness was determined by ISO standard 15184:2012 by a pencil test.

Specimens including coated and uncoated oak were investigated. The chemical groups of the samples were examined by Fourier transform infrared spectroscopy (FTIR, BRUKER Vertex 70v, Hamburg, Germany) equipped with an attenuated total reflection (ATR) accessory. The surfaces were put in contact with the ZnSe crystal at a 45° angle of incidence.

The contact angle of pure oak with testing liquid (distilled water) and solution of 70 wt% PUA coating was measured according to ISO 15989:2004 by a contact angle meter (ISO, 2004; Krüss K11MK4, Hamburg, Germany). The contact angles of coated wood with different coating amounts with distilled water was also measured.

The surface morphologies were captured by atomic force microscopy (AFM) and collected by a scanning probe microscope in contact mode. The scanning area was 2 μm × 2 μm. The surface roughness was tested by a surface roughness tester using a stylographic method (SF200 Basic, Beijing Shidaishanfeng Technology Co. Ltd., Beijing, China). The values of the average profile error (Ra), maximum height (Rp), maximum valley depth (Rv), maximum profile height (Rt) and Maximum peak-valley height (Rz) were obtained.

Energy dispersed X-ray analysis (EDXA, Horiba 7021-H, Tokyo, Japan) was carried out in SEM analysis mapping mode. The PUA coated samples were cut vertically. The image and distribution of elemental N, mainly from the PUA resin, was captured digitally to allow further analysis of the penetration of PUA into each substrate.

## 3. Results and Discussion

### 3.1. Morphologies

**Figure 1:**
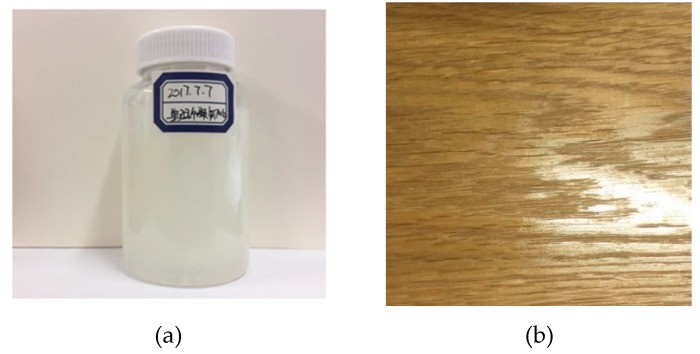

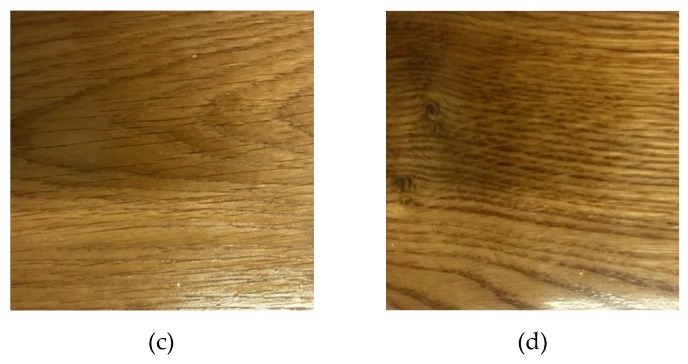
Images of PUA coating and its coated oak samples at different coating amounts. (**a**) PUA coating; (**b**) 12 g/m^2^ PUA coated oak; (**c**) 60 g/m^2^ PUA coated oak; (**d**) 120 g/m^2^ PUA coated oak.

**Figure 2:**
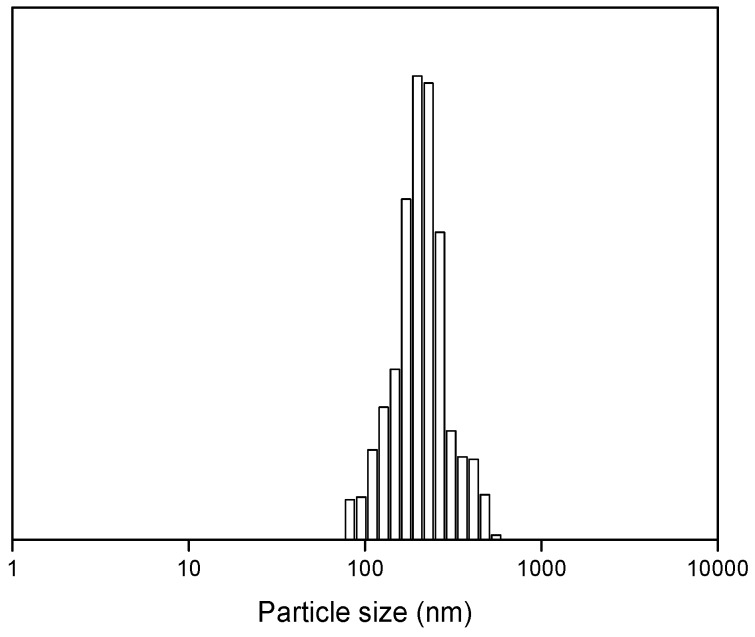
Particle size distribution of the PUA coating.

Figure 1 shows images of the fast water-based UV curing PUA coating and the coated wood samples. It can be seen that the synthesized PUA coating was a stable and transparent liquid. Table 2 lists some parameters of the fast water-based UV curing PUA coating, such as the solid content, viscosity, and average particle size. It can be found that the PUA coating had very high solid content of 90 wt%, while the viscosity was not very high. The particle size of the PUA coating ranged from 80 nm to 500 nm (Figure 2) with an average value of 226 nm. Images of PUA coated oak are seen in Figure 1b–d. From the images, all wood samples were well coated by the PUA coating with a layer of coating film. 

**Figure 3:**
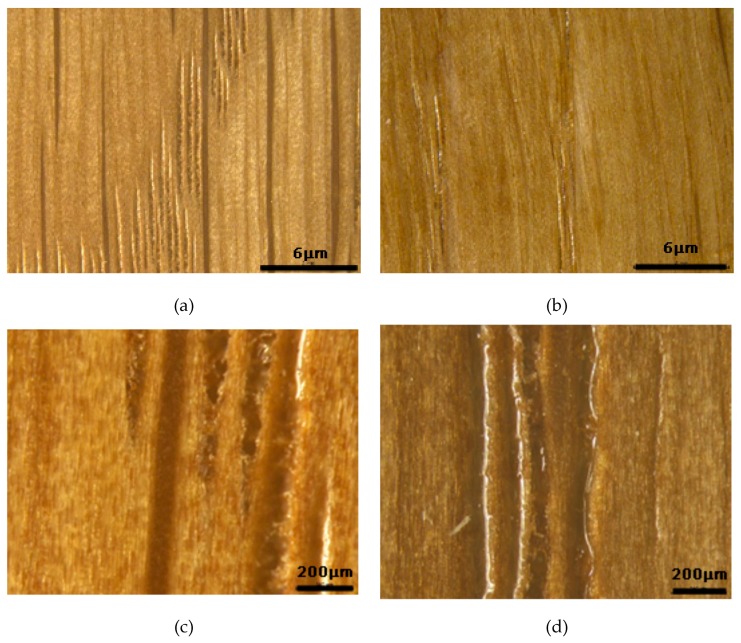
Optical microscope images of oak after coating at different coating amounts. (**a**,**c**) 12 g/m^2^ PUA coated oak; (**b**,**d**) 60 g/m^2^ PUA coated oak.

Figure 3 shows a visual microscope picture of the oak surface with different coating amounts under an optical stereomicroscope. The wood samples were covered by the coating films. Besides, it can be seen that as the coating amount increased, the surface of the paint film became smoother. When the coating amount was 12 g/m^2^, the oak vessels were partly occupied by paint, but most regions were still exposed. When the coating amount increased to 60 g/m^2^, the vessels were substantially filled with few defects. 

**Figure 4:**
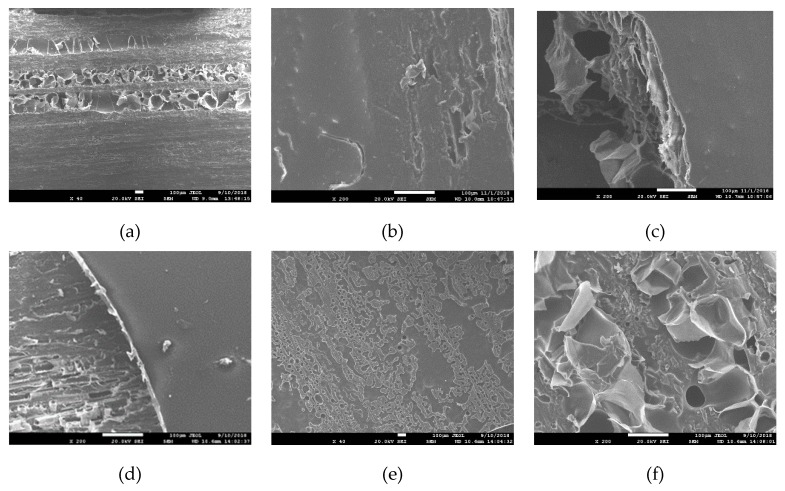
SEM images of oak before and after coating at different coating amount. (**a**) pure oak; (**b**) 12 g/m^2^ PUA coated oak; (**c**) 60 g/m^2^ PUA coated oak; (**d**) 120 g/m^2^ PUA coated oak; (**e**,**f**) the back surface of coating peeling from the oak; (**a**,**e**) the magnification was ×40; (**b**–**d**,**f**) the magnification was ×200.

The surface morphologies of oak under different coating amounts tested by SEM are shown in Figure 4. The microstructures of the oak were clearly visible before painting. After the surface was coated with PUA coating, the microstructures were covered to varying degrees at different coating amounts. Within a certain range, as the coating amount increased, the surface smoothness of the sample gradually increased, which was consistent with the roughness values (discussed in the following part). In the case of 12 g/m^2^ coated samples, the coating surface was incompletely covered, and some oak microstructures were exposed, namely, the surface of the painted film had a large number of grain protrusions, and the surface was rough. As the coating amount increased, the surface coverage of the oak increased, where the oak vessels could not be seen. 

In order to further understand the adhesion between PUA coating and wood, the painted film was peeled off from the test piece, and the morphology of the back was observed (Figure 4e,f). It can be seen from the figure that the back of the paint film exhibited a microscopic topography similar to that of oak. However, some tyloses were easily found, which may be caused by the adhesion of coating in the vessels during drying of the water.

From the morphology results, it can be concluded that the PUA coating can form a layer of film that covers on the surface of the oak after 22 min of curing. Besides, we tested the drying state of all coatings according to ISO 9117:1990, and found no damage or markings on the surfaces. Therefore, the coatings were completely dried and be cured on oak by this process, which is much faster than the Chinese standard requirement for water-based coatings of 30–60 min (Table 3).

Besides, the PUA coating has good adhesion to wood, because the back surface was similar to the morphology of wood. Therefore, the preparation and application of the fast water-based UV curing PUA wood coating was successful.

### 3.2. Basic Properties of PUA Coated Oak

The basic properties including dried coating thickness, adhesion, hardness, and gloss of the PUA coated oak samples are listed in Table 4. As the coating amount increased, the dried thickness, adhesion, hardness, and gloss of the sample were improved. By comparing with dried coating thickness values, it seemed that the dried coating thickness did not increase linearly. For example, at a coating amount of 120 g/m^2^, the dried coating thickness was 74 μm, which was 21 times the dried coating thickness at 12 g/m^2^. It may be that the surface of the sample was not completely covered at the coating amount of 12 g/m^2^, and more PUA molecules were penetrated into the wood substrate, resulting in a low adhesion, hardness and surface gloss of the paint film. When the coating amount increased to 60 g/m^2^, the coverage was improved, therefore, the adhesion classification, hardness, and surface gloss of the sample were improved. When the coating amount was 120 g/m^2^, the sample was completely covered with the paint film, where the adhesion classification reached level 1 with the hardness of 2H and the gloss of 92.56°. 

By comparing with the technical requirements for some related wooden products given by Chinese and international standards, listed in Table 3, it can be inferred that at 60 g/m^2^, the adhesion classification and pencil hardness of the PUA coating met the requirements for water-based coatings and UV curing coatings. Besides, the curing time is much less than the requirement of water-based coatings of 30–60 min. For wooden products, all PUA coated samples can be used for wooden furniture and wood-based wallboard. However, for indoor wood-based doors and solid wood floors, the coating amount should increase to 60 g/m^2^, while for applying on engineered wood floors, the coating amount must be further increased to 120 g/m^2^.

### 3.3. Chemical Analysis

**Figure 5:**
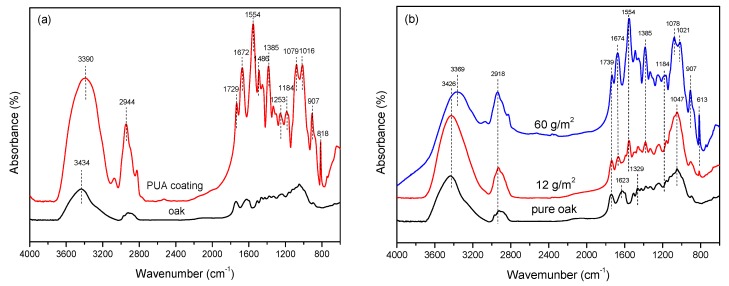
ATR-FTIR spectrum of samples. (**a**) Pure oak and PUA coating; (**b**) oak before and after coating with different coating amounts.

Figure 5a shows the ATR-FTIR results for oak and pure PUA coating. The characteristic absorption bands attributed to the polyurethane-acrylate coating are reflected in the figure based on the literature [11,14,16]. The peaks observed at 1554 cm^−1^ and 818 cm^−1^ were assigned to –NH in and out of plane bending vibrations, which indicated that N element was in the coating. Therefore, the penetration depth of the coating can be determined according to the N element. In addition, bands at 1184 cm^−1^, 1079 cm^−1^, and 907 cm^−1^ were attributed to the C–O stretching vibrations, C–O stretching vibrations in ether bonds, and acid –OH out-of-plane bending vibrations, respectively. These indicated that carboxylic acid functions may be present in the coating, which can be reacted with the hydroxyl groups in the wood. Meanwhile, bands at 1726 cm^−1^ and 1672 cm^−1^ are characteristic peaks of carbonyl C=O and C=O stretching vibrations in unsaturated acids. Besides, other characteristic peaks of PUA can be observed, such as the 1251 cm^−1^ C–O stretching vibration in ester bonds, 1016 cm^−1^ C–OH stretching vibrations, 1385 cm^−1^ –OH in-plane bending vibrations, and 3390 cm^−1^ –OH stretching vibration.

Figure 5b shows the FTIR results of pure oak and oak coated with PUA at amounts of 12 g/m^2^ and 60 g/m^2^, respectively. The comparison shows that the PUA characteristic peak gradually becomes obvious with the increase of the coating amount. However, some characteristic peaks shifted compared with the PUA spectrum shown in Figure 5a. Compared with the oak, 12 g/m^2^ and 60 g/m^2^ coated samples owned many characteristic peaks, such as the characteristic absorption peak of C=O stretching vibrations in unsaturated acids at 1674 cm^−1^, the absorption peak of –NH_2_ stretching vibrations at 1554 cm^−1^, and the 1184 cm^−1^ C–O stretching vibration. These vibration absorption peaks indicated that the PUA coating covered the surface of the oak and exhibited the characteristic PUA absorption peaks. As the coating amount increased, in the FITR of oak coated with 60 g/m^2^ PUA one can clearly observe that some chemical groups changed compared with that coated with 12 g/m^2^ PUA, indicating that some chemical reactions took place. In details, the absorption peaks at 1047 cm^−1^ disappeared, while C–O stretching vibrations in ether bonds and C–OH stretching vibrations appeared at 1078 cm^−1^ and 1021 cm^−1^. Additionally, the characteristic –OH out-of-plane bending vibration peaks at 907 cm^−1^, –CH_2_/CH_3_ stretching vibration peaks at 2918 cm^−1^, –OH internal bending vibration at 1385 cm^−1^, –CH_2_ out-of-plane bending vibrations at 613 cm^−1^, and out-of-plane bending vibrations at 818c m^−1^ were obviously enhanced, showing the characteristics of PUA coating. Compared with pure PUA, the –OH absorption peak shifted to 3369 cm^−1^, and the 1021 cm^−1^ C–OH stretching vibration absorption peak was weakened, while the stretching vibration at 1184 cm^−1^ for C–O was enhanced. This proved that the hydroxyl groups in the wood reacted with the coating by hydrogen bonds or by forming esters [34].

### 3.4. Contact Angle

**Figure 6:**
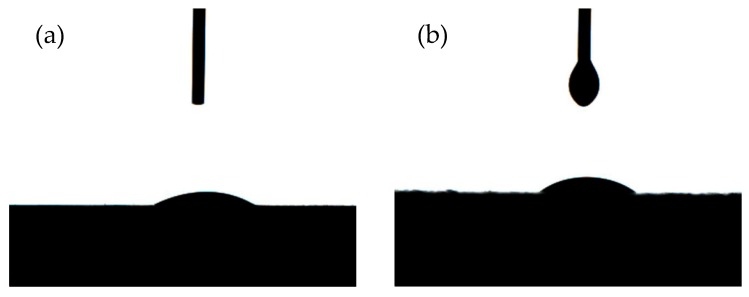

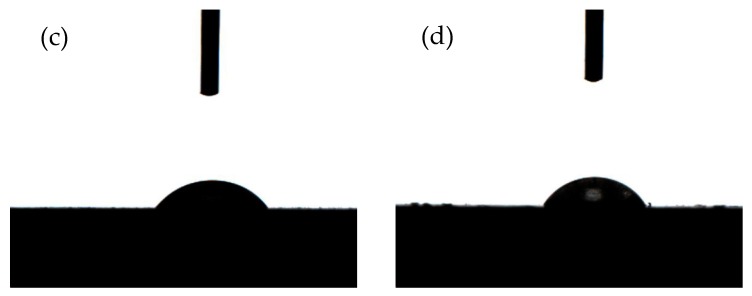
Contact angles of oak sample. (**a**) Pure oak with water; (**b**) pure oak with 70 wt% PUA droplet; (**c**) 12 g/m^2^ PUA coated oak with water; (**d**) 60 g/m^2^ PUA coated oak with water.

Molecular interactions between the wood surface and the coating are also important in achieving good adhesion [35]. Figure 6 shows the image of contact angle of PUA droplets (70% solids) and water on the oak surface. The results are listed in Table 5. The coating is not easily spread on the oak compared to water because the polarity of PUA is lower than that of water. Although the wettability of PUA is not as good as that of water, the contact angle of PUA droplets on oak was lower than 90°, indicating the PUA can evenly spread on the oak surface. The contact angle between PUA coated oak and water were also tested. The results showed that with the increase of coating amount, the contact angle of water droplets on the surface increased because of the covering of a layer of coating film. Compared with oak coated at 12 g/m^2^ PUA amount, the 60 g/m^2^ possessed a larger contact angle with water, which could be explained by the incompletely covered surface at 12 g/m^2^ PUA amount, where the water might penetrate into the wood sample.

### 3.5. Surface Roughness

Table 6 shows the surface roughness of oak coated with different coating amounts. As the coating amount increased, Ra decreased continuously, and all the surface roughness values showed the same tendency. The Rp and the Rv decreased with the increase of the coating amount, indicating that the vessels and surface irregularities of the oak were continuously filled by PUA coating. Thus, the roughness values decreased, and the surface became smooth.

**Figure 7:**
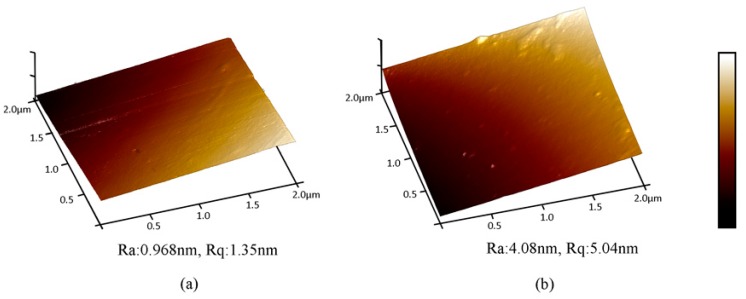
AFM images of oak after coating at different coating amount. (**a**) 12 g/m^2^ PUA coated oak; (**b**) 60 g/m^2^ PUA coated oak.

The surface morphologies of the oak after coating at different amounts tested by AFM are shown in Figure 7. It can be seen from the figure that as the coating amount increased, the surface coverage of the oak increased, and the PUA coating can cover well on the oak under different coating amounts without obvious defects. Among them, when the coating amount was 12 g/m^2^, the surface roughness value was 0.97 nm. As for the surface with a coating amount of 60 g/m^2^, many small protrusions were observed, and the surface paint film was rough after drying, and the surface roughness value was 4.08 nm. Different from Table 5, it can be seen that the roughness of the coating increased with increasing coating amount, which might because that the paint surface was uneven, resulting in an increase in the surface roughness in a very small range.

### 3.6. Penetration of PUA in Wood

**Figure 8:**
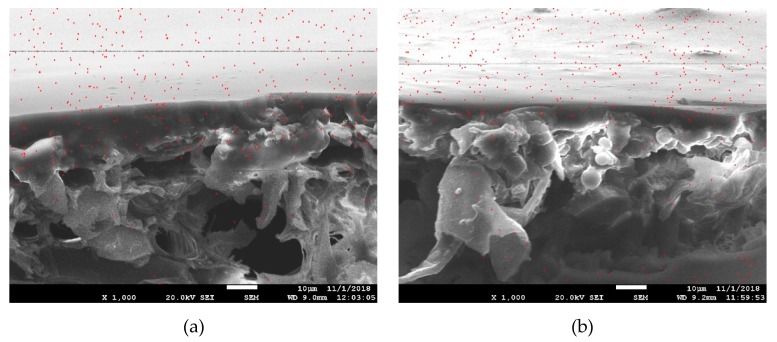
SEM-EDXA images for N distribution of oak after coating at different coating amount. (**a**) 12 g/m^2^ PUA coated oak; (**b**) 60 g/m^2^ PUA coated oak; the magnification was ×1000.

In order to further analyze the permeability of different coatings in oak, the SEM-EDXA was performed on N-element (see red dots) for the coated samples. The results are shown in Figure 8. As seen from the figure, the PUA was distributed in a large amount on the surface of the substrate, and a small amount was penetrated into the substrate. At the coating amount of 12 g/m^2^, the PUA penetration depth on the oak surface was about 20 μm, and the small portion reached 35 μm. When the coating amount increase to 60 g/m^2^, the voids in the oak penetration depth of 35 μm are substantially filled because of higher amount of PUA was applied. However, the penetration of the PUA coating in oak did not increase significantly, and 35 μm was the limit.

## 4. Conclusions

The preparation and application of the fast water-based UV curing PUA wood coating was successful, where it can be completely cured within 22 min. During curing, the hydroxyl groups in the oak reacted with the coating. It can be seen that as the coating amount increased, the number of hydroxyl groups decreased and the contact angle increased. At the same time, within a certain range, the adhesion, hardness and gloss value of oak surface increased to different extents. The coating coverage was improved and the surface gradually became smooth. The PUA can reach a depth of 35 μm from the wood surface, and with the increase of coating amount, the filling degree of PUA coating in wood increased.

## Figures and Tables

**Table 1:** Reagents for water-based UV curing PUA coating.

Reagent	Function	Manufacturer
Ammonium persulfate (APS)	Initiator	1
Acrylic acid (AA)	Monomer	1
Styrene (ST)	Monomer	1
Ethyl acrylate (EA)	Monomer	1
Hydroxyethyl acrylate (HEA)	Monomer	1
Toluene-2,4-diisocyanate (TDI)	Monomer	1
Polyethylene glycol (Mn: 400) (PEG-400)	Monomer	1
Dibutyltin dilaurate (DBTD)	Catalyst	1
N-methyl-2-pyrrolidone	Solvent	1
Hexanediol diacrylate (HDDA)	Diluting agent	1
γ-Aminopropyl triethoxysilane (KH550)	Silane	2
Polyether siloxane copolymer composition	Defoamer agent	3
2-Hydroxymethylphenylpropan-1-one	Photoinitiator	4

*Notes*: (**1**) Xilong Chemical Co.Ltd (Guangzhou.China); (**2**) Qufu Chengguang Chemical Co., Ltd (Shandong, China); (**3**) TEGO AIREX 901W; (**4**) Germany Evonik tego.

**Table 2:** Parameters of fast water-based UV curing PUA coating.

Parameter	Values
Solid content	90 wt%
Viscosity	50 mpa·s
Average particle size	226 nm

**Table 3:** Technical requirements for coatings and the related wooden products.

Technical requirements	Standards	Adhesion classification	Pencil hardness	Curing time (min)
Water-based coatings for woodenware for indoor decorating and refurbishing	GB/T 23999-2009	≤1	≥B	30–60
	EN 927:2006	≤1	--	--
UV curing coatings for woodenware	HG/T 3655–2012	≤2	≥H	--
Wooden furniture	GB/T 3324–2017	≤3	--	--
	CEN/TS 16209:2011	≤2	--	--
Solid wood floor	GB/T 15036–2018	≤3	≥H	--
	ISO 17959:2014	≤3	≥H	--
Engineered wood floor	GB/T 18103–2013	≤2	≥2H	--
	EN 14354–2005	≤2	--	--
Indoor wood-based door	LY/T 1923–2010	≤2	≥HB	--
Wood-based wall-board	LY/T 1697–2017	≤2	≥2B	--

**Table 4:** Adhesion, hardness, and gloss of oak coated at different coating amounts.

Sample	Dried coating thickness (μm)	Adhesion classification	Pencil hardness	Gloss (°)
12 g/m^2^ PUA coated oak	3	2	B	28.7
60 g/m^2^ PUA coated oak	34	1	H	83.2
120 g/m^2^ PUA coated oak	74	1	2H	93.6

**Table 5:** Contact angles of PUA droplets (70% solids) and water on the oak surface.

Sample	Testing liquid	Contact angle (°)
Pure oak	Water	29.68
Pure oak	70 wt% PUA droplet	34.98
12g/m^2^ PUA coated oak	Water	52.22
60g/m^2^ PUA coated oak	Water	56.13

**Table 6:** Roughness of oak before and after coating at different coating amount.

Sample	Ra (μm)	Rp (μm)	Rv (μm)	Rt (μm)	Rz (μm)
Pure oak	3.456	5.930	12.521	55.110	18.450
12 g/m^2^ PUA coated oak	3.026	3.451	10.000	45.975	13.451
60 g/m^2^ PUA coated oak	2.422	1.651	6.924	48.010	8.577
120 g/m^2^ PUA coated oak	0.310	0.516	0.400	2.834	0.916
